# Supplementary material for: Rapid turnover of life-cycle-related genes in the brown algae
Source: Genome Biol. 2019 Feb 14;20:35. doi: 10.1186/s13059-019-1630-6 (PMC6374913; doi:10.1186/s13059-019-1630-6)
Supplement: Supplementary file 3 — Protein alignments, corresponding Gblocks html file and CDS sequences for the lineage conserved gametophyte-biased genes. (ZIP 2109 kb) [file 13059_2019_1630_MOESM3_ESM.zip › Con_GA_alignments/Ec-21_004310.aa_ali.fasta-gb.htm]

Ec-21\_004310.aa.fasta.trans.aa\_ali.fasta


## Gblocks 0.91b Results

Processed file: **Ec-21\_004310.aa.fasta.trans.aa\_ali.fasta**  
Number of sequences: **2**  
Alignment assumed to be: **Protein**  
New number of positions: **334** (selected positions are underlined in blue)

```
                         10        20        30        40        50        60
                 =========+=========+=========+=========+=========+=========+
Ec-21_004310     MKNIYHGTTLLKLLHHPHSVSFRPHAVQKTRSWSNIAQAFTEKKRIRPASAPAIKHKEHQ
MSTRG.58040.1    ------------------------------------------------------------
                                                                             


                         70        80        90       100       110       120
                 =========+=========+=========+=========+=========+=========+
Ec-21_004310     TQPSVDSGSSRGRAETARARLTDGTTRRDGPMSFQRAEEIQEVVDVLQHTMMARLRGRPK
MSTRG.58040.1    ------------------------------------------------------------
                                                                             


                        130       140       150       160       170       180
                 =========+=========+=========+=========+=========+=========+
Ec-21_004310     KGERKKAAEAAKRAGSAPGTSVVDRRSHGHAPRVASPRGSAPATSSRGAGADRRRAASGA
MSTRG.58040.1    ------------------------------------------------------------
                                                                             


                        190       200       210       220       230       240
                 =========+=========+=========+=========+=========+=========+
Ec-21_004310     SGEQRRRRPRPSTAWNHRELPARCPPAIPTGMEHGLKARPKTAAVIASSFSKSKRSLAED
MSTRG.58040.1    ------------------------------------------------------------
                                                                             


                        250       260       270       280       290       300
                 =========+=========+=========+=========+=========+=========+
Ec-21_004310     LGPLRWSPGLIGDESHKTDTDGKLGMTRARIYSFGASTQGGGGHEEDMLRATAIDAARRS
MSTRG.58040.1    ------------------------------------------------------------
                                                                             


                        310       320       330       340       350       360
                 =========+=========+=========+=========+=========+=========+
Ec-21_004310     RLPRRCSSAGSAPRDLRPAVPGRRETGEDSVRDDKDGGGGSAAGGYGGVKGRYGNASCRA
MSTRG.58040.1    ------------------------------------------------------------
                                                                             


                        370       380       390       400       410       420
                 =========+=========+=========+=========+=========+=========+
Ec-21_004310     GADSVHEDSTDSSEHLGVVDISKGDDLSWLLMFFLSPFSRQLTTVLSHAEVSGYIRATGG
MSTRG.58040.1    ------------------------------------------------------------
                                                                             


                        430       440       450       460       470       480
                 =========+=========+=========+=========+=========+=========+
Ec-21_004310     KHSSFTSGSKLKGGGGGVASMSAIRGSPLLADGIANKGTSPAALLDNGGGSGNDEEAPTV
MSTRG.58040.1    ------------------------------------------------------------
                                                                             


                        490       500       510       520       530       540
                 =========+=========+=========+=========+=========+=========+
Ec-21_004310     PETSSWSGSSSATESDSGGNDDDTEQRESADRSGSDTRTETRGRSDRGREVGTRTVAGTA
MSTRG.58040.1    ------------------------------------------------------------
                                                                             


                        550       560       570       580       590       600
                 =========+=========+=========+=========+=========+=========+
Ec-21_004310     ENEGVMPTGSAPPQLDCLRGRYARADCKSKSAYAIKKAAAATAVGAAGASTTAPQTPHVL
MSTRG.58040.1    ------------------------------------------------------------
                                                                             


                        610       620       630       640       650       660
                 =========+=========+=========+=========+=========+=========+
Ec-21_004310     TSATRCNKRGLFDRSGARDRGGRGGGRGGGRGARDPASWRDRFAAVQRRRAVLDDLRRTE
MSTRG.58040.1    -------------------------------------------AAVQRRRAILEEAMRSE
                                                            #################


                        670       680       690       700       710       720
                 =========+=========+=========+=========+=========+=========+
Ec-21_004310     ATRQVKARAKAEARVPNNASVAASVSVRGAKRFTTLDGGAGNNGNATEKNKNNMTININS
MSTRG.58040.1    ANRLVEACAERGARSLCNASVSVSVCVRGVD--DVLDS------------KAR------S
                 #############################                               


                        730       740       750       760       770       780
                 =========+=========+=========+=========+=========+=========+
Ec-21_004310     NKSAPNPASYRTVQAAVTVQRAFRSTLQYEAWLGDQSDRDYLSQVTLRDGDGKRDEPEPS
MSTRG.58040.1    D-EGTRPLFKRKVEAAMMIQRAFRASRQRELWLGNQCDRDYASQVTP-EKNNVEDDGQGV
                       ########################################        ######


                        790       800       810       820       830       840
                 =========+=========+=========+=========+=========+=========+
Ec-21_004310     HHQSSALKRSPSSAGGADSIISRLNTVRSDEWAAHRAKKLHDRHVTIRLKRMVRGFICCT
MSTRG.58040.1    IQDSTQRLQLPTGLG-TEPSTSFLGASH-----PFKTWKLRRRHVAIRTKRMIRGFISCT
                 ###############                       ######################


                        850       860       870       880       890       900
                 =========+=========+=========+=========+=========+=========+
Ec-21_004310     QARFLLLQLLWDRTEGNILGERRSKRAAVESRAASTVQQLSHIRKLAVGLIKIQNSAEAF
MSTRG.58040.1    QARFLLLQLLWDKTEGIILGERRSKRAAVESRAAVTIQQLSHIRRIAVGLIKMQSSAQSF
                 ############################################################


                        910       920       930       940       950       960
                 =========+=========+=========+=========+=========+=========+
Ec-21_004310     KLEQQARQAKRNPKKTAKPSGDKHGGRHQGGGGGRVFDTAKQARSEKAECEAILREHGRR
MSTRG.58040.1    KLEHQSRPAKRGTKTNPRGVRQNQGARNQGGGG-CAFETAKEARSEKAECEAILREHGRR
                 ##############                      ########################


                        970       980       990      1000      1010      1020
                 =========+=========+=========+=========+=========+=========+
Ec-21_004310     GRRLVDIAVRDRFLRDLLYSHRRQHIIRDQREYLMETSRPPLVDSRDVKRMLECPAEEMH
MSTRG.58040.1    GRRLVDVAVRDRFLRDLLYSFRRQHITRDHSEYLLETSRPPFVDSRDVKQMLACPAEEMH
                 ############################################################


                       1030      1040      1050      1060      1070      1080
                 =========+=========+=========+=========+=========+=========+
Ec-21_004310     MFSDPMNYRTRPRYTRLALPFREIGGIIQDTIASNVRSEYRDKLKEALGPTDARESGIHQ
MSTRG.58040.1    LFSDPKNYRTRPRYTRLALPLREIEGRIQVNDKK--------------------------
                 #############################                               


                       1090      1100      1110      1120      1130      1140
                 =========+=========+=========+=========+=========+=========+
Ec-21_004310     QEPYSSPAGAFAEDTNTGWVHKDATAAAAQQPRQQQQAPTAEHAHKNSNKTRASVNFDTG
MSTRG.58040.1    ------------------------------------LAPACCRAPWLSTSSRCEV-----
                                                      ##################     


                       1150      1160      1170      1180      1190      1200
                 =========+=========+=========+=========+=========+=========+
Ec-21_004310     AAATTTPPHQQRAAVGMLAAGHKPTPAVLAPQTATAVAAAASRSAVQGRAWRSGRDGAAM
MSTRG.58040.1    ----------------------------------------------VHLHFE---ARIVH
                                                                             


                       1210
                 =========+==
Ec-21_004310     AVPMMTAKATAA
MSTRG.58040.1    AVPYWSPPRQTQ
```

```
Parameters used
Minimum Number Of Sequences For A Conserved Position: 2
Minimum Number Of Sequences For A Flanking Position: 2
Maximum Number Of Contiguous Nonconserved Positions: 8
Minimum Length Of A Block: 10
Allowed Gap Positions: None
Use Similarity Matrices: Yes
```

```
Flank positions of the 6 selected block(s)
Flanks: [644  689]  [727  766]  [775  795]  [819  914]  [937  1049]  [1118  1135]  

New number of positions in Ec-21_004310.aa.fasta.trans.aa_ali.fasta-gb:  334  (27% of the original 1212 positions)
```
